# Beta-Adrenergic Blockade in Critical Illness

**DOI:** 10.3389/fphar.2021.735841

**Published:** 2021-10-15

**Authors:** Rebecca Bruning, Hannah Dykes, Timothy W. Jones, Nathaniel B. Wayne, Andrea Sikora Newsome

**Affiliations:** ^1^ Department of Clinical and Administrative Pharmacy, University of Georgia College of Pharmacy, Augusta, GA, United States; ^2^ Department of Pharmacy, Augusta University Medical Center, Augusta, GA, United States

**Keywords:** beta-blockers, critical illness, sepsis, esmolol, tachyarrhythmia, hemodynamics

## Abstract

Catecholamine upregulation is a core pathophysiological feature in critical illness. Sustained catecholamine β-adrenergic induction produces adverse effects relevant to critical illness management. β-blockers (βB) have proposed roles in various critically ill disease states, including sepsis, trauma, burns, and cardiac arrest. Mounting evidence suggests βB improve hemodynamic and metabolic parameters culminating in decreased burn healing time, reduced mortality in traumatic brain injury, and improved neurologic outcomes following cardiac arrest. In sepsis, βB appear hemodynamically benign after acute resuscitation and may augment cardiac function. The emergence of ultra-rapid βB provides new territory for βB, and early data suggest significant improvements in mitigating atrial fibrillation in persistently tachycardic septic patients. This review summarizes the evidence regarding the pharmacotherapeutic role of βB on relevant pathophysiology and clinical outcomes in various types of critical illness.

## Introduction

The catecholamine cascade is a defining element of critical illness (Preiser et al., 2014). The α- and β-adrenergic receptors form the response mechanism for endogenous catecholamines and exogenously administered catecholamine vasoactive agents (e.g., dobutamine, dopamine, norepinephrine, and epinephrine) (Preiser et al., 2014). These receptors elicit responses in nearly every major organ system and change their expression levels during the body’s stress response to critical illness (Belletti et al., 2020). Prolonged exposure to high levels of catecholamines in these altered states may evoke detrimental metabolic and hemodynamic effects. Higher levels of catecholamines appear in a myriad of critical illness etiologies and have been associated with higher mortality (Tripathi et al., 1981; Hamill et al., 1987; Benedict and Rose, 1992; Boldt et al., 1995; Dunser and Hasibeder, 2009).

β-blockers (βB) may be administered to manipulate the adrenergic response during critical illness. βB are mainstay medications for cardiovascular disease states, including post-myocardial infarction management (O'Gara et al., 2013), atrial fibrillation (AF) (January et al., 2014), and heart failure (HF); however, evaluation of the utility of βB extends beyond long-term cardiac management into acute management of critically ill patients (Writing et al., 2021). The purpose of this review is to critically evaluate available literature regarding βB therapy in critical illness and describe evidence-based βB use in presentations of critical illness including sepsis, severe burns, traumatic brain injury, and cardiac arrest.

## Methods

A literature search was performed to identify studies including critically ill patients who received βB therapy. The PubMed database was searched for studies published between January 1970 and March 2021 using combinations of the search terms beta-blockers, cardiac arrest, critical illness, esmolol, intensive care unit, sepsis, septic shock, severe burns, and traumatic brain injury. Studies reporting on patients managed in the intensive care unit (ICU) setting treated with βB were included. No limits on study designs were made and included prospective, retrospective, observational, or interventional designs. References within original research articles, review articles, editorials, abstracts, meta-analyses, and systematic reviews were screened for inclusion. A summary of the included works outlining sample size, disease state, βB agent used, dosing, timing of initiation, and outcomes can be found in Table 1. Furthermore, a summary of frequently questions regarding βB in critical illness are summarized in Table 2.

**TABLE 1:** Dosing and timing of βB in critical illness.

*Sepsis*	Population	β-Blockade	Initiation	Outcome
Study				
Schmittinger et al. (2008) *Retrospective*	40 patients with septic shock and cardiac depression	Metoprolol 25–47.5 mg PO Increased gradually to achieve target HR (65–95 bpm) (n = 40)	Initiated only after stabilization of cardiovascular function (17.7 ± 15.5 h after shock onset or ICU admission)	HR control was achieved in 97.5% of patients (n = 39) within 12.2 ± 12.4 h HR, CVP, and norepinephrine, vasopressin, and milrinone dosages decreased (all *p* < 0.001) CI remained unchanged whereas SVI increased (*p* = 0.002)
Gutierrez et al. (2009) *Retrospective*	83 septic patients	Any βB exposure (n = 54) vs. no exposure (n = 29) Dosing not reported	Not reported	βB not significantly associated with mortality in the univariate (OR = 1.83; 95% CI = 0.59–5.69) nor multivariate model (OR = 1.843; 95% CI = 0.56–6.10)
Berk et al. (1972) *Case series*	26 patients with refractory septic shock and SBP <70 mm Hg and UOP <12 ml/h	Propranolol 5 mg given over 2–3 h period followed by another 5 mg during the next 6–12 h (n = 11)	Approximately 24–48 h from start of shock. Considered refractory to all conventional interventions (fluids, antibiotics, steroids)	Increased BP, PaO2, urinary output, and total peripheral resistance in before and after propranolol use case series Decreased CVP, CO, and HR Survival resulted in the 8 who had a normal or increased CO prior to βB. The 3 who did not survive had very low CO
Gore and Wolfe (2006) *Prospective*	6 moderately septic, mechanically ventilated patients with pneumonia	Esmolol infusion to target 20% HR reduction (range: 6–22 mg/min) (n = 6)	Infusion started immediately following 5 h basal measurements	Significant decrease in CI (*p* < 0.05) proportional to decrease in HR (*p* < 0.05) No significant difference in SVR, SVI, BP, extremity/hepatic blood flow, REE, oxygen consumption
Balik et al. (2012) *Prospective*	10 septic patients	Esmolol bolus (0.2–0.5 mg/kg) followed by continuous 24 h infusion with titration to achieve 20% decrease of baseline HR (n = 10)	After correction of preload (2 h after sepsis)	HR decreased from mean 142 ± 11/min to 112 ± 9/min (*p* < 0.001) Insignificant reduction of CI (4.94 ± 0.76 to 4.35 ± 0.72 L/min/m2). SV insignificantly increased. No significant changes of norepinephrine infusion (0.13 ± 0.17 to 0.17 ± 0.19 μg/kg/min), DO2, VO2, OER or arterial lactate
Morelli et al. (2016) *Pilot*	45 septic shock patients, with an HR ≥ 95 bpm and requiring norepinephrine to maintain MAP ≥65 mmHg	Titrated esmolol infusion to maintain HR between 80 and 94 bpm (n = 45)	≥24 h after hemodynamic optimization	Decreased HR Decrease in Ea Decreased SV (all *p* < 0.05) CO, EF unchanged NE requirements were reduced (*p* < 0.05)
Shang et al. (2016) *Prospective*	151 patients with severe sepsis	Esmolol infusion initial dose 0.05 mg/kg/min adjusted to target HR 70–100 bpm (n = 75) vs control (n = 76)	Not reported	HR reached target within 72 h for both treatment groups ScvO2 increased in the esmolol group and decreased in the control group (*p* < 0.01). Lactate reduction in control group at 48 h (*p* < 0.05) Shorter duration of mechanical ventilation in the esmolol group (*p* < 0.05)
Du et al. (2016) *Prospective*	63 patients with septic shock within 48 h of diagnosis	Esmolol 20 mg loading dose following by 25 mg/h infusion to achieve HR reduction by 10–15% from baseline (n = 63)	Hemodynamically stable with HR ≥ 100 bpm <48 h after septic shock started	BP was unaltered SV was increased compared with before esmolol therapy (43.6 ± 22.7 vs. 49.9 ± 23.7 ml; *p* = 0.047) Decreased lactate levels (1.4 ± 0.8 vs. 1.1 ± 0.6 mmol/L; *p* = 0.015)
Morelli et al. (2013) *RCT*	154 septic patients	Esmolol 25 mg/h (titrated every 20 min to reach target HR 80–94 bpm) (n = 77) vs control (n = 77)	Initiated after randomization that was performed after resuscitation with fluid and vasopressors for 24 h	Decreased HR—28 bpm [IQR −7−21; *p* < 0.001] Decreased NE requirement −0.01 [IQR −0.2–0.44; *p* = 0.003] Decreased 28-days mortality 49.4 vs. 80.5% (*p* < 0.001)
Yang et al. (2014) *RCT*	41 septic patients	Esmolol 0.05 mg/kg/min (adjusted to achieve HR of <100 bpm in 2 h) (n = 21) vs. control (n = 20)	Initiated after randomization that was performed after 6-h resuscitation with fluid and vasopressors	Decreased HR 12 h (93 ± 4; *p* < 0.05); Decreased CI (3.3 ± 0.8; *p* < 0.05) No significant changes in MAP, CVP, or SVI ScVO2 was not decreased
Wang et al. (2015) *RCT*	90 septic patients	Esmolol + milrinone (n = 30) vs. milrinone (n = 30) vs. control (n = 30) Dosing not reported	Not reported	100% HR control (74–94 bpm) within 96 h of initiation (*p* < 0.001 vs. milrinone) Increased 28-days survival 60 vs. 33.3% (milrinone) vs. 26.7% (control) Decreased NE use 0.07 ± 0.04
Xinqiang et al. (2015) *RCT*	48 septic patients	Esmolol 0.05 mg/kg/min (adjusted to achieve HR of <100 bpm within 24 h) (n = 24) vs. control (n = 24)	Initiated after randomization that was performed after resuscitation with fluid and vasopressors for 6 h	Decreased LOS (13.75 ± 8.68 vs. 21.7 ± 6.06; *p* < 0.001) Decreased 28-days mortality (25.0 vs. 62.5%; *p* < 0.009) Decreased HR, arterial lactate levels Increased SVRI, SVI, ScVO2 (all *p* < 0.01)
Wang et al. (2017) *RCT*	76 septic patients	Esmolol 0.05 mg/kg/hr (titrated every 5 min to reach the HR of <95/min within 4 h) (n = 30) vs. control (n = 30)	Initiated after randomization that was performed after resuscitation with fluid and vasopressors for 24 h	HR decreased significantly at each time point No significant difference in MAP CI significantly increased at > 24 h SVI significantly increased at > 4 h No difference in 28-days mortality (30 vs. 36.7%; *p* = 0.583)
Liu et al. (2019) *RCT*	100 septic patients	Esmolol 25 mg/h (titrated every 20 min to reach the HR between 80 and 100/min within 12 h) (n = 50) vs control (n = 50)	Initiated after randomization that was performed after being resuscitated with fluid and vasopressors for 24 h	No difference in 28-days mortality (62 vs 68%; *p* = 0.529) Lower HR on day 1–7; but overall no statistically significant difference in HR (*p* > 0.05) No significant difference in total does of NE, lactate level, inflammatory markers, APACHEⅡ, SOFA, hospital LOS (all *p* > 0.05)
Kakihana et al. (2020) *RCT*	151 septic patients with HR > 100 bpm and diagnosis of atrial fibrillation, atrial flutter, or sinus tachycardia	Landiolol 1 μg/kg/min (titrated every 15–20 min, until the HR decreased to less than 95 bpm) (n = 76) vs. control (n = 75)	Landiolol was initiated within 2 h after randomization that was conducted after being resuscitated with fluid and vasopressors (mean time from ICU admission to randomization: 15.8 h in landiolol vs. 13.5 h in control)	Larger proportion of patients had HR 60–94 bpm 24 h after randomization (55% [41 of 75] vs. 33% [25 of 75]), with a between-group difference of 23.1% (95% CI 7.1–37.5; *p* = 0.0031) Decreased incidence of new-onset arrhythmia by 168 h (9 vs. 25%; *p* = 0.015) No significant difference in 28-days mortality (*p* = 0.22), hospital free days (*p* = 0.91), ICU free days (*p* = 0.55), and ventilator free days (*p* = 0.47)
Walkey et al. (2016) *Retrospective*	39,693 septic patients with atrial fibrillation	CCB (n = 14,202) vs. βB (IV metoprolol, esmolol, atenolol, labetalol, and propranolol; n = 11,290) vs. digoxin (n = 7,937) vs. amiodarone (n = 6,264)	On average, received first atrial fibrillation medication 1–2 days into hospital stay	βB were associated with lower hospital mortality when compared with CCBs (n = 18,720; relative risk [RR] = 0.92; 95% CI = 0.86–0.97), digoxin (n = 13,994; RR = 0.79; 95% CI = 0.75–0.85), and amiodarone (n = 5,378; RR = 0.64; 95% CI = 0.61–0.69) Results were similar among subgroups with new-onset or preexisting AF, heart failure, vasopressor-dependent shock, or hypertension
Bosch et al. (2020) *Retrospective*	666 septic patients with atrial fibrillation with rapid ventricular response	CCB (n = 225) vs. βB (IV metoprolol or esmolol; n = 67) vs. amiodarone (n = 337) vs. digoxin (n = 37)	Amiodarone and CCB added within 1–2 h of start of atrial fibrillation vs 4.9 h for digoxin vs. 10.2 h for βB	The adjusted hazard ratio for HR of <110 beats/min by 1 h was 0.50 (95% CI = 0.34–0.74) for amiodarone vs. βB, 0.37 (95% CI = 0.18–0.77) for digoxin vs. βB, and 0.75 (95% CI = 0.51–1.11) for CCB vs. βB
Macchia et al. (2012) *Retrospective*	9,465 septic patients	Chronic outpatient βB (n = 1,061) vs. no previous βB treatment (n = 8,404)	N/A Pre-morbid βB	Lower mortality at 28 days (188/1,061 [17.7%]) than those previously untreated (1857/8,404 [22.1%]) (OR = 0.78; 95% CI = 0.66–0.93; *p* = 0.005)
Fuchs et al. (2017) *Prospective (secondary analysis)*	296 septic patients with chronic βB treatment	Continuation of βB during acute phase of sepsis (n = 167) vs. discontinuation during sepsis (n = 129)	Acute phase of sepsis defined as 2 days before to 3 days after disease onset	Continuation of βB therapy was significantly associated with decreased hospital (*p* = 0.03), 28-days (*p* = 0.04) and 90-days mortality rates (40.7 vs. 52.7%; *p* = 0.046)
Singer et al. (2017) *Retrospective*	6,839 septic patients	Chronic outpatient βB (n = 2,838) vs. no previous βB treatment (n = 4,001)	N/A Pre-morbid βB	Decreased mortality during hospitalization (24 vs 31%; *p* < 0.0001) Multivariable logistic regression models 31% decrease in in-hospital mortality (adjusted OR = 0.69; CI = 0.62–0.77) Decreased 30-days mortality (13 vs. 18%; *p* < 0.0001)
Guz et al. (2021) *Prospective*	1,186 septic patients	Chronic outpatient βB (n = 320) vs no previous βB treatment (n = 866)	N/A Pre-morbid βB	No significant difference in crude 30-days and 90-days mortality rates (30 days, 15 vs 19% [*p* = 0.25]; 90 days, 22 vs 24% [*p* = 0.51]) Reduction in 30-days mortality rates for patients with absolute tachycardia (OR = 0.406; 95% CI = 0.177–0.932) 30-days survival benefit in the subgroup of patients with relative tachycardia in both univariate and multivariate analysis (OR = 0.496; 95% CI = 0.258–0.955; *p* = 0.04)
***Burns***				
**Study**	**Population**	**Beta-blockade**	**Initiation**	**Outcome**
Baron et al. (1997) *Prospective*	22 pediatric burn patients (>40% of TBSA)	Propranolol 0.5–1 mg/kg PO or IV Q 8 h for 10 days (n = 22)	During the catecholamine-induced hypermetabolic phase	Propranolol use significantly decreased daily average HR (10–13%) and RPP (10–16%) compared to 24-h mean pre-treatment
Herndon et al. (2001) *RCT*	25 pediatric burn patients (>40% of TBSA)	Propranolol 0.33 mg/kg/4 h through NGT (n = 13) vs. control (n = 12) (dose later adjusted for HR 20% less than basal)	Propranolol was initiated immediately following the second staged grafting procedure (approximately 8–12 days after initial admission)	Propranolol decreased HR (*p* = 0.001) decreased REE (*p* = 0.001), oxygen consumption (*p* = 0.002), and prevented lean mass loss (*p* = 0.01)
Jeschke et al. (2007) *RCT*	245 pediatric burn patients (>40% of TBSA)	Propranolol 0.5–1.5 mg/kg/6 h PO (n = 102) vs. control (n = 143)	Started after 7 days	No significant difference between groups in terms of mortality (5 vs. 6%), incidence of infections (21 vs. 30%), or sepsis (7 vs. 10%) Decreased REE (*p* < 0.05)
Herndon et al. (2012) *RCT*	179 pediatric burn patients (>30% of TBSA)	Propranolol dose required to reduce HR 15% (mean dose 4 mg/kg/day PO) (n = 90) vs control (n = 89)	Propranolol started 3 ± 2 days after admission	Propranolol reduces HR (*p* = 0.01), cardiac work, central body mass and trunk fat, and improves lean body mass and bone mineral density (*p* = 0.02) Decreased likelihood of total body mass loss at 6 months (OR = 0.5; 95% CI = 0.25–0.75) No difference in mortality (*p* = 0.72)
Williams et al. (2011) *RCT*	406 pediatric burn patients (>30% of TBSA)	Propranolol 1 mg/kg/day PO (divided Q 6 h; adjusted for HR 15–20% less than basal) (n = 171) vs. control (n = 235)	From 24 to 72 h until end of admission (once patients were fluid stabilized)	Propranolol at dose of 1 mg/kg/day reduces HR 15% with respect to basal The dose must increase to 4 mg/kg/day the first 10 days in order to maintain the effect (*p* < 0.05)
Arbabi et al. (2004) *Retrospective*	129 adult burn patients (mean TBSA 14 ± 12%); 21 pre-hospital βB vs 22 hospital βB vs. 86 control	Metoprolol, atenolol, esmolol, labetalol, or propranolol (at therapeutic doses)	All pre-hospital βB patients remained on treatment once admitted Hospital βB patients were initiated on βB a mean 8.8 days postinjury	In multivariate analyses, pre-hospital βB use was associated with significant decrease in fatal outcome and healing time (5 vs 13% control; *p* < 0.05)
Mohammadi et al. (2009) *RCT*	79 adult burn patients (20–50% of TBSA)	Propranolol 1 mg/kg/d and max dose of 1.98 mg/kg/d given in six divided doses (adjusted to achieve 20% HR reduction from baseline) (n = 37) vs. control (n = 42)	Started on 4th day of admission after hemodynamic stabilization	Decreased healing time (16.13 ± 7.40 days vs. 21.52 ± 7.94 days; *p* = 0.004) Less time required before skin grafting procedure (28.23 ± 8.43 days vs. 33.46 ± 9.17 days; *p* = 0.007) Decreased size of burn wound that needed grafting (*p* = 0.006) Shorter hospital LOS (30.95 ± 8.44 days vs. 24.41 ± 8.11 days; *p* = 0.05)
Ali et al. (2015) *RCT*	69 adult burn patients (>30% of TBSA)	Propranolol at a dose that reduces HR by 20% (average dose 3.3 ± 3.0 mg/kg/day) (n = 35) vs. control (n = 34)	Administered within 48 h of burn and given throughout hospital stay	Lower daily average HR over 30 days (*p* < 0.05) Decreased blood loss during grafting procedures (5–7% improvement in perioperative hematocrit; *p* = 0.002) Decreased time between grafting procedures (10 ± 5 days vs. 17 ± 12 days; *p* = 0.02)
Cheema et al. (2020) *RCT*	70 adult burn patients (20–40% of TBSA)	Propranolol at dose of 0.5–3 mg/kg/day (adjusted to achieve a 20% max HR reduction) (n = 35) vs. control (n = 35)	Started on 3rd postburn day after hemodynamic stabilization	Less muscle wasting (mean mid-arm circumference 27.57 ± 1.62 cm vs. 24.46 ± 1.77 cm; *p* < 0.0001) Faster wound healing (13.20 ± 1.90 days vs 20.34 ± 2.32 days; *p* < 0.001) Less time required before skin grafting procedure (23.87 ± 2.36 vs. 33.64 ± 3.15 days; *p* < 0.001) Shorter hospital LOS (26.69 ± 3.58 days vs 37.71 ± 3.68 days; *p* < 0.001)
***Traumatic Brain Injury (TBI)***				
**Study**	**Population**	**Beta-blockade**	**Initiation**	**Outcome**
Cruickshank et al. (1987) *RCT*	114 patients with acute head injury	Atenolol 10 mg IV Q 6 h for 3 days followed by atenolol 100 mg PO once daily for 4 days (n = 56) vs control (n = 58)	Immediately after hemodynamic stabilization (mean 20.2 h after trauma)	Significantly inhibited the rise in arterial CKMB (*p* < 0.01) Abolished focal myocardial necrotic lesions Reduced likelihood of SVT and ST-segment and T-wave changes
Arbabi et al. (2007) *Retrospective*	4,117 trauma patients with and without head injury	βB therapy (n = 303) vs. control (n = 3,814)	Administration of scheduled βB during the hospital stay	Significantly decreased risk of mortality in all patients (OR = 0.3; *p* < 0.001) and patients with severe head injury (OR = 0.2; *p* < 0.001) No significant difference in late deaths after 48 h of hospitalization (OR = 0.7; *p* = 0.2)
Cotton et al. (2007) *Retrospective*	420 patients with a head Abbreviated Injury Scale ≥3	Metoprolol, propranolol, labetalol, atenolol, esmolol, or sotalol use (n = 174) vs. control (n = 246)	Administration of βB for at least 2 consecutive days during hospitalization	Significantly decreased mortality rate (*p* = 0.036)
Inaba et al. (2008) *Retrospective*	1,156 patients with blunt head injuries requiring ICU admission	βB therapy (n = 203) vs control (n = 953)	Administration of βB during hospitalization in the ICU	Significantly decreased overall mortality rate (adjusted OR = 0.54; 95% CI = 0.33–0.91; *p* = 0.01) Significantly decreased mortality rate in patients ≥55 years old with severe head injuries (28 vs. 60%; OR = 0.3; 96% CI = 0.1–0.6; *p* = 0.001)
Schroeppel et al. (2010) *Retrospective*	2,601 patients with blunt TBIs	Atenolol, carvedilol, esmolol, labetalol, metoprolol, nadolol, propranolol, or sotalol use (n = 506) vs. control (n = 2,095)	Administration of more than one dose of a βB during hospitalization	Decreased mortality rate (OR = 0.347; CI = 0.246–0.490; *p* < 0.0001)
Schroeppel et al. (2014) *Retrospective*	1,755 patients with TBIs	Atenolol, carvedilol, esmolol, labetalol, metoprolol, propranolol, or sotalol (n = 427) vs. control (n = 1,328) Propranolol (n = 78) vs. other βB (n = 349)	Administration of more than one dose of a βB during hospitalization	No difference in mortality rate between βB and control with the adjusted analysis (adjusted OR = 0.850; 95% CI = 0.536–1.348) Decreased mortality rate with propranolol compared to other βB (3 vs 15%; *p* = 0.002)
Zangbar et al. (2016) *Retrospective*	356 patients with blunt TBIs requiring ICU admission	Metoprolol (n = 178) vs. no βB (n = 178)	Administration of at least one dose of a metoprolol during hospitalization in the ICU	Significantly decreased mortality rate (78 vs 68%; *p* = 0.04) No difference in the mean heart rate (*p* = 0.99)
Mohseni et al. (2015) *Retrospective*	874 patients with an isolated severe TBI and an intracranial injury with Abbreviated Injury Scale ≥3	Labetalol, metoprolol, or other βB (n = 287) vs. control (n = 587)	Administration of a βB during hospitalization with median time to first admission of 1 day and 75% of patients receiving the first dose by day 3	Significantly decreased mortality rate (11 vs 17%; *p* = 0.007) Significantly increased mortality rate in patients not on pre-hospitalization βB (adjusted OR = 3.0; 95% CI = 1.2–7.1; *p* = 0.015)
Ko et al. (2016) *Retrospective*	440 patients with a moderate to severe TBI (head Abbreviated Injury Scale 3–5) requiring ICU admission	Propranolol 1 mg IV Q 6 H within 24 h of admission while in the ICU, then 40 mg PO BID after patient transferred to the floor (n = 109) vs. control (n = 331)	Administration of propranolol within 24 h of admission	Significantly decreased mortality rate after predictors of mortality were adjusted (adjusted OR = 0.25; *p* = 0.012)
Murry et al. (2016) *Retrospective*	38 patients with moderate to severe TBI requiring ICU admission	Early low dose propranolol 1 mg IV Q 6 H (n = 28) vs. standard of care, which could include βB (labetalol, metoprolol) at any point during hospitalization (n = 10)	Administration of propranolol within 12 h of ICU admission and for a minimum of 48 h	Decreased rates of bradycardia events (1.6 vs. 5.8; *p* = 0.05) Decreased rates of hypotensive events (0.8 vs. 0.5; *p* = 0.6) Decreased ICU LOS (15.4 vs. 30.4 days; *p* = 0.02) and hospital LOS (10 vs. 19.1 days; *p* = 0.05) Similar mortality rates (10 vs. 10.7%; *p* = 0.9)
Ley et al. (2018) *Prospective*	2,252 patients with TBI requiring ICU admission	Atenolol, esmolol, propranolol, metoprolol, labetalol, or another βB (n = 1,120) vs. control (n = 1,132)	Administration of βB during hospitalization	Decreased 30-days mortality rate (13.8 vs 17.7%; *p* = 0.013) Decreased 30-days mortality rates with propranolol vs. other βB (9.3 vs. 15.9%; *p* = 0.003) Increased hospital LOS (21 ± 25 days vs 10 ± 37 days; *p* < 0.01) Increased hospital LOS with propranolol vs. other βB (21 ± 25 days vs. 13 ± 14 days; *p* < 0.01)
***Cardiac Arrest***				
**Study**	**Population**	**Beta-blockade**	**Initiation**	**Outcome**
Lee et al. (2016) *Retrospective*	41 patients with RVF in out-of-hospital cardiac arrest	Esmolol (loading dose: 500 μg/kg, infu- sion: 0–100 μg/kg/min) (n = 16) vs control (n = 25)	Given after obtaining verbal informed consent from patient’s proxies, written consent afterwards	Significantly more sustained ROSC (56 vs 16%; *p* = 0.007) Increased survival and good neurological outcomes at 30 days, 2 months, and 6 months (18.8 vs. 8%; *p* = 0.36)
Driver et al. (2014) *Retrospective*	25 patients with RVF in out-of-hospital cardiac arrest	Esmolol (loading dose: 500 μg/kg, infu- sion: 0–100 μg/kg/min) (n = 6) vs control (n = 19)	Approximately 46 min into cardiac arrest (range 34–59 min)	Higher rates of temporary (67 vs. 42%) and sustained ROSC (67 vs. 32%) Increased survival to ICU admission (66 vs. 32%) and discharge (50 vs. 16%) Increased discharge with favorable neurologic outcome (50 vs. 11%) No stats are significant given small sample size
Nademanee et al. (2000) *Prospective*	49 patients with frequent VF/VT episodes with recent MI	Propranolol IV 0.15-mg/kg dose over 10 min and then as a 3–5-mg dose Q 6 h (n = 14) vs Esmolol IV 300–500-mg/kg loading dose for 1 min followed by maintenance dose of 25–50 mg/kg/min (n = 7) vs LSGB (n = 6) vs. antiarrhythmic (n = 22)	Received sympathetic blockade treatment within 1 h after all of the antiarrhythmic medications initiated during the code were discontinued	Decreased mortality significantly at 1-week (22 vs. 82%; *p* < 0.0001) and 1 year (67 vs. 5%; *p* < 0.0001) compared to antiarrhythmic medication
Chatzidou et al. (2018) *Prospective*	60 ICD patients with recurrent VF/VT within a 24-h period	Propranolol 40 mg PO Q 6 h (cumulative dose 160 mg/24 h) (n = 30) vs Metoprolol 50 mg PO Q 6 h (cumulative dose 200 mg/24 h) (n = 30)	Not documented	Propranolol patients had decreased incidence of VT/VF (*p* = 0.001) and decreased ICD discharges (*p* = 0.004) More propranolol patients were free of arrhythmic events within 24 h (90 vs 53.3%; *p* = 0.03) Arrhythmic events were more likely to be terminated with propranolol (hazard ratio = 0.225; 95% CI = 0.112–0.453; *p* < 0.001) Time to arrhythmia termination and hospital LOS were significantly shorter with propranolol compared to metoprolol (*p* < 0.05 for both)
Skrifvars et al. (2003) *Retrospective*	98 patients receiving post-resuscitation care within 72 h of out-of-hospital VF arrest (79 βB vs 19 control)	Metoprolol (at least 50 mg PO BID or 5 mg IV BID) or bisoprolol (at least 2.5 mg two times a day orally) n breakdown not reported	Initiated within 72 h post-resuscitation	Increased survival in multiple regression model (44 vs 79%; *p* = 0.005)
***KEY***				
APACHE II = acute physiology and chronic health evaluation	βB = beta-blockers	BID = twice daily	BP = blood pressure	
CCB = calcium channel blocker	CI = cardiac index, confidence interval	CKMB = myocardial isoenzyme of creatine kinase	CO = cardiac output	
CVP = central venous pressure	DO2/VO2 = systemic oxygen delivery/consumption	Ea = static arterial elastance	EF = ejection fraction	
HR = heart rate	ICD = implantable cardioverter defibrillator	ICU = Intensive Care Unit	IV = intravenous	
LOS = length of stay	LSGB = left stellate ganglionic blockade	MAP = mean arterial pressure	MI = myocardial infarction	
N/A = not applicable	NGT = nasogastric tube	NE = norepinephrine	OER = oxygen extraction ratio	
OR = odds ratio	PaO2 = arterial oxygen pressure	PO = oral	REE = resting energy expenditure	
ROSC = return of spontaneous circulation	RPP = rate pressure product	R/VF = refractory ventricular fibrillation	SBP = systolic blood pressure	
ScVO2 = central venous oxygen saturation	SOFA = sequential organ failure assessment	SV = stroke volume	SVI = stroke volume index	
SVR = systemic vascular resistance	SVRI = systemic vascular resistance index	SVT = supraventricular tachycardia	TBSA = total body surface area	
VT = ventricular tachycardia				

**TABLE 2:** Practical questions regarding βB use in critical illness.


**1. Are βB safe in critical illness?** Yes, βB appear to be safe in the setting of critical illness. Adequate volume resuscitation should be a target prior to βB initiation to ensure appropriate preload
**2. What are the hemodynamic effects of βB in critical illness?**
**a. HR:** reduce heart rate
**b. SV:** decreased inotropy is expected; however, in a patient with adequate preload, increased diastolic times may improve filling and improve SV
**c. CO:** decreased inotropy and chronotropy are expected effects; however, due to potential increases in SV/cardiac efficiency, βB effect on CO can be neutral to improved
**d. MAP:** Blood pressure is the product of CO and systemic vascular resistance (SVR). βB have no notable effects on SVR, but potential improvements in CO can be observed, especially in the setting of mitigating arrhythmias (e.g., atrial fibrillation). As such, cardioselective βB use may be associated with neutral to positive effects on MAP.
**3. How does βB use effect vasoactive agents like norepinephrine?**
When used at the appropriate time (i.e. if persistent tachycardia remains despite fluid resuscitation and control of pain and agitation), βB can be norepinephrine-sparing allowing for decreases in norepinephrine dosages without a higher need for inotropic support. βB allow decreased HR which facilitates increased ventricular filling times during diastole, subsequently increasing SV, SVR, and left ventricularLV stroke work to maintain MAP and lower catecholamine requirements
**4. Should pulmonary conditions like COPD or asthma preclude βB use in critically ill patients?** In the setting of a compelling indication (e.g., atrial fibrillation), no, βB should not be withheld due to this co-morbidity. Further, continuation of home βB use even in the setting of pulmonary conditions appears safe and associated with improved outcomes. In particular, cardioselective βB (e.g., esmolol) appear to be the lowest risk
**5. How should βB be dosed in different types of critical illness?**
**a. Sepsis:** Data are mixed; however, esmolol 0.5 mg/kg/min or 25 mg/h IV continuous infusions are the two most frequent published approaches. In most studies, infusions were titrated to achieve a 20% HR reduction
**b. Burns:** Dosing ranges of propranolol 0.5–3 mg/kg/day IV or PO divided three to four times per day were most prevalent in the existing literature
**c. TBI:** A wide variety of agents and doses have been studied with most robust data reporting use of atenolol, esmolol, propranolol, metoprolol, or labetalol, but failing to mention dosing strategies
**d. Cardiac Arrest:** IV esmolol loading doses were reported as 300–500 μg/kg as well as 300–500 mg/kg. Propranolol, metoprolol, and bisoprolol were also utilized

### Beta-Adrenergic Physiology and Rationale in Critical Illness

**FIGURE 1:**
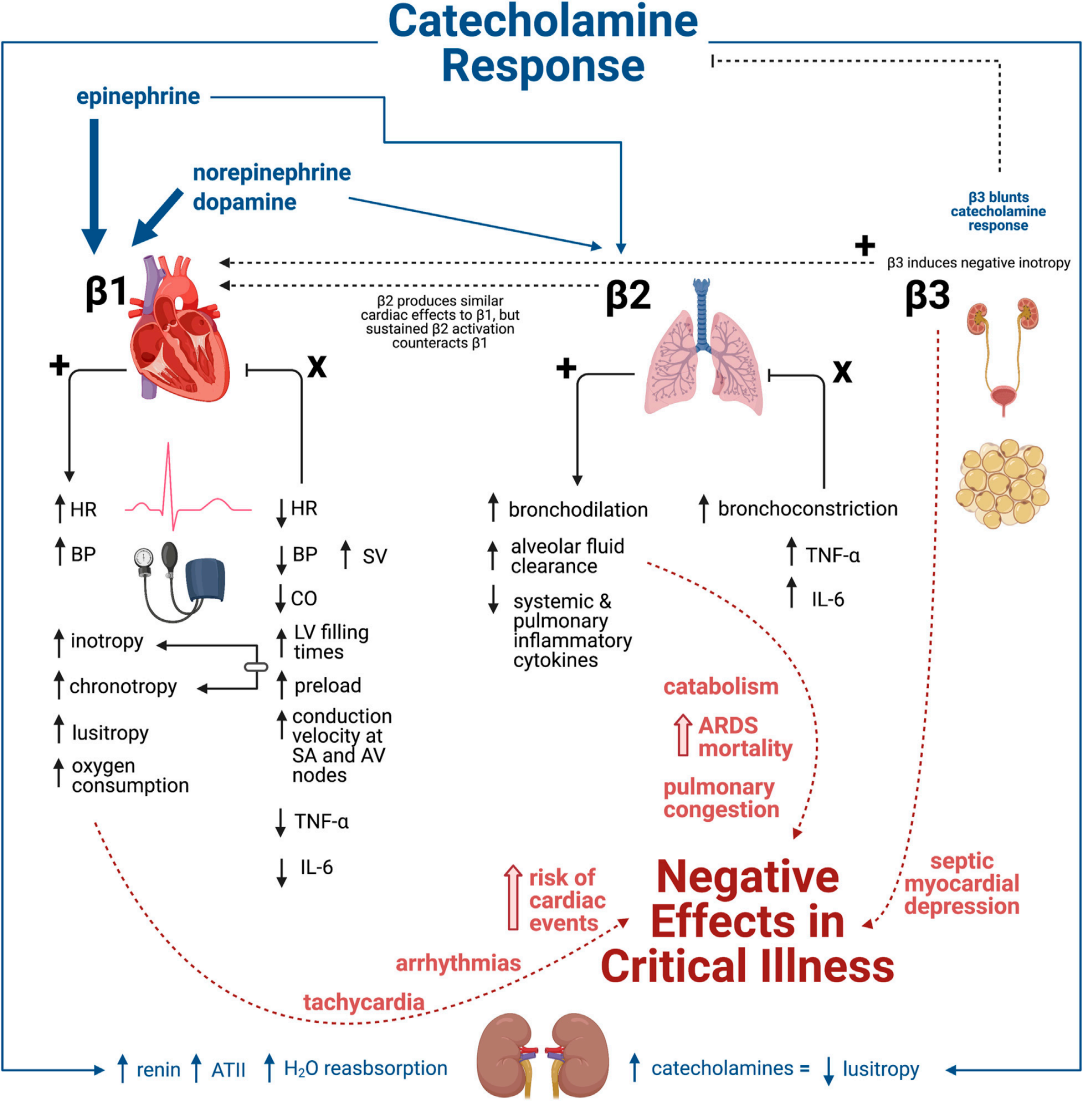
Physiologic response to β-adrenergic receptor agonism and antagonism in critical illness. The catecholamine response characterized by epinephrine, norepinephrine, and dopamine release result in stimulation of β1 (majorly) and β2 (minorly). In contrast, β3 agonism blunts the catecholamine response. The physiologic response to β1, β2, and β3 agonism culminates in numerous negative effects within critical illness that can ultimately lead to negative clinical outcomes including increased mortality. This introduces the beneficial physiologic response of βB antagonism as a way to mediate the detrimental effects of the hyperadrenergic state prominent in various types of critical illness. Illustration created with BioRender.com.

To appreciate the potential benefits of βB as a pharmacologicallyy supported intervention inhas several rationales for use during critical illness, conceptualization of β-adrenergic physiology during critical illness is necessary. Alterations in both as the dysregulated signaling molecules and receptorsadrenergic cascade provides intervenable pathways. Complexity further increases as the detrimental adrenergic susceptibilities differ among organ systems, withThese β-adrenergic effects in are pronounced in cardiac and pulmonary tissues most relevant to critically ill patientsduring a critically ill state (Dunser and Hasibeder, 2009). Figure 1 provides a visual representation ofillustrates the physiologic response to β receptor agonism and antagonism, emphasizing the negative effects of β receptor stimulation in critical illness.

#### Catecholamine Up-Regulation

The principle sSympathetic nervous system (SNS) signaling hormones include the catecholamines norepinephrine, (agonist of α1, α2, β1, and minorly β2), epinephrine (agonist of α1, α2, β1, and β2), and dopamine, which increase during any state of stress (dose-dependent agonist of α1, α2, β1, and minorly β2) (Table 3). (Dunser and Hasibeder, 2009) The stress of Ccritical illness results in massive SNS signaling (Dunser and Hasibeder, 2009). Cardiac arrest and septic shock display profound increases in circulating epinephrine (up to 300 times baseline) and norepinephrine (14 times baseline) (Wortsman et al., 1984; Jones and Romano, 1989). High These higher circulating catecholamine levels are associated with increased mortality. and may potentially be used as an additional factor in predicting mortality These higher circulating catecholamines can predict mortality in the critically ill (Benedict and Rose, 1992; Boldt et al., 1995), but wWhether catecholamine upregulation represents treatable pathophysiology or a necessary compensation inevitably linked to disease severity and poorer outcomes remains debatedshould be targeted remains unclear, as it provides the physiologic adaptation to shock and critically ill states.

**TABLE 3:** Adrenergic receptor selectivity of endogenous catecholamines.

Catecholamine	α1	α2	β1	β2	DA1	DA2
Epinephrine	+++	+++	+++	+++	−	−
Norepinephrine	+++	+++	++	+	−	−
Dopamine 0–3 μg/kg/min	−	+	−	−	+++	++
2–10 μg/kg/min	+	+	++	+	++	++
>10 μg/kg/min	++	++	++	+	++	++

DA = dopaminergic receptor.

Adapted from Table 2 in Dunser et al. (Dunser and Hasibeder, 2009)

#### Cardiac β Effects

β-adrenergic pathways extensively regulate cardiac function and function and, specifically, hemodynamics due to extensive cardiac expression. β1 comprises 80% of cardiac β-receptors and mediates inotropy, chronotropy, lusitropy (i.e. relaxation rate), and dromotropy (i.e. conduction speed). However, at high concentrations of catecholamines, the lusitropic effect is overwhelmed by tachycardia and increased contractility (Dunser and Hasibeder, 2009; Wachter and Gilbert, 2012). β2 produces similar cardiac effects to β1 (Belletti et al., 2020), but sustained β2 activation leads to a counteracting of β1 effects (Lucia et al., 2018). Moreover, the cardiac-mediated epinephrine response appears independent of functional β2 and is mediated primarily by β1relies on β1 activation (Chruscinski et al., 1999). This Experimental evidence touts β1 activation appearspathways as proapoptotic to cardiac myocytes, while β2 may confer protection (Patterson et al., 2004). Although, interestingly, recent preclinical data in mice demonstrated prevention of cardiac mitochondrial dysfunction via ablation of β2 signaling after burns (El Ayadi et al., 2019). β1 predominates cardiac expression over β2 (4 to 1), but states such as HF can tip the balance nearly even through β1 downregulation by sustained adrenergic stimulation (Bristow et al., 1986). Increased β2 expression may provide benefits through increase contractility and angiogenesis, Research diverges in identifying cardioprotective vs. deleterious roles from the higher proportion of β2 expression as some reports indicate improvements in contractility, angiogenesis, and cardiac remodeling (Rengo et al., 2012). but In contrast, others implicatemay promote β2 as arrhythmiasmogenic (Nguyen et al., 2015). However, commonly used transgenic mice strains overexpressing β2-receptors may represent non-physiologic environments given that HF does not upregulate β2-receptors. This augmented receptor physiology may increase the rate of arrhythmogenicity attributed to β2-receptors in these animal studies (Bristow et al., 1986). Unlike β1 and β2, β3 induces negative inotropy and blunts the catecholamine response (Moniotte et al., 2001), and expression is upregulated in critical illness (Moniotte et al., 2007). IThe increased β3 expression may prime the heart for consequences like septic myocardial depression (Yang et al., 2018). Interestingly, Myagmar et al. recently described the absence of β2 and β3 in cardiac myocytes, while β1 was present in all myocytes. β2 and β3 are primarily in other cell types (e.g., endothelial cells) underscoring the reliance on β1 in cardiac muscle, which raise further concerns regarding the appropriateness of artificial β2 overexpression in cardiac myocytes (Myagmar et al., 2017).

Clinical evidence supporting harmful β-mediated harmful effects has been reported. β1 drives Ttachycardia in critical illness (primarily β1 driven) may increase the, increasing the risk of cardiac events in those with pre-existing heart disease (Sander et al., 2005). Additionally, left ventricular (LV) apical ballooning syndrome (i.e., Takotsubo syndrome) has a links to endogenous adrenergic stimulation (Wittstein et al., 2005) and specifically β-agonism (Hajsadeghi et al., 2018). In sepsis, despite elevated catecholamines, overall β-receptor downregulation contributes to septic myocardial dysfunction (Suzuki et al., 2017). Cumulatively, these cardiac effects support the rationale of study behindfor β1-selective βB (e.g., esmolol) in critical illness, as β2 and β3 are is potentially protective.

#### Cardiac β-Blockade Effects

Antagonism The antagonism of cardiac β-receptors produces negative inotropic and chronotropic effects asslows conduction velocity through sino-atrial and atrioventricular nodes and produces negative inotropic and chronotropic effects decreases. These effectsThis mechanism may decrease cardiac output and blood pressure, demonstrated by. Experimental studies have shown impairment ined right ventricular function and worsened perfusion with βB when useduse at the onset of septic shock (Coppola et al., 2015). Typically, these effects limit their use in critical illness application; h. However, experimental assessments are often limited by short observation times (several hours) compared to more extended follow-up in clinical studies that would evaluate judicious use of βB after acute hemodynamic stabilization. Preclinical studies have suggested positive effects as beta-blockade with agents selective for β1 agents may reduced tumor necrosis factor alpha (TNF-α) and interleukin 6 (IL-6) in the serum and myocardiumsystemic and cardiac inflammation (Suzuki et al., 2005; Hagiwara et al., 2009). HoweverIn contrast, antagonism of β2 increases TNF-α and IL-6inflammation and may exacerbate the physiologic changes seen inof sepsis (Lang et al., 2008), further supporting β1 selective benefits.

**FIGURE 2:**
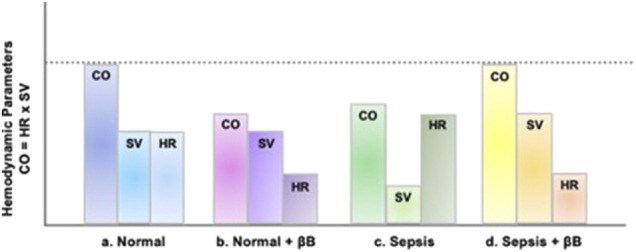
Hemodynamic effects of sepsis and β-Blockade. In panel A, the stroke volume (SV) and cardiac output (CO), stroke volume (SV), and heart rate (HR) of a normal, healthy individual are presented. In panel B, due to the negative inotropy associated with βB, which causes reduced HR, the overall CO is reduced despite normal SV. In panel C, sepsis results in tachycardia, due to excessive sympathetic activation. This increase in HR does not allow for adequate ventricular filling causing a decrease in CO secondary to a decrease in SV. In panel D, given that venous return (i.e., preload) is adequate, then βB-induced HR reduction allows for more left ventricular filling time, subsequently decreasing afterload and increasing SV enough to overcome decreased HR and improved CO.

The hemodynamic benefits of βB may improve cardiac function in critical illness through may occur through increased left ventricula LVr filling times by reducing heart rateas heart rate (HR) lowers and there is enhanced ventricular-arterial (V-A) coupling (Mathieu et al., 2016). Patients with septic shock experience a V-A decoupling associated with poor LV function (Guarracino et al., 2014). Morelli et al. demonstrated β1 selective esmolol reduced arterial elastance, and increased stroke volume, reduced with esmolol (a β1 selective βB) heart rate in septic shock reduction, suggesting improved V-A coupling (Morelli et al., 2016). Further, esmolol increased stroke volume (SV) in septic shock despite decreases in cardiac output (CO). Figure 2 describes the hemodynamic effects of sepsis and concomitant βB. βBThese effects are attributable to the reductions in heart rateHR reductions, enhancing end-diastolic filling of the left ventricleLV and thus increasingto increase preload. No differences in oxygenation and tissue perfusion were noted despite decreased CO (Du et al., 2016). Experimentally, esmolol protects myocardial function in sepsis, likely through mitigating apoptotic pathways in the myocardium that are associated with elevated β1 stimulation (Herndon et al., 2001; Wang et al., 2017). Indeed, esmolol added to cardioplegic solutions for cardiac surgery reduced post-surgery troponins suggesting cardiac tissue preservation (Bignami et al., 2017). In acute decompensated HF, continuation of chronic βB appears to prevent death (Prins et al., 2015; Jones et al., 2020). While it may seem logical to stop negative inotropes in patients hospitalized with a failing heart, discontinuation of βB did not significantly affect hemodynamics in these patients (Butler et al., 2006). A meta-analysis of βB effects in septic shock trials supports neutral hemodynamic effects after initial resuscitation despite vasopressor support requirements after initial resuscitation (Lee et al., 2019). Taken together, these The extrapolation of preclinicalpreclinical and clinical data support beneficial, or at least safe, cardiac and hemodynamic effects cardiac βB during critical illness data. to clinical settings support beneficial effects, although clinical studies lack details discerning if these suggested mechanisms are the drivers of clinical benefit.

#### Pulmonary β Effects

The pulmonary vasculature has modest concentrations of β-receptors. Within the lungs, β2 -receptors are the most consequential in the lungs as they outnumber β1 three to one in most pulmonary tissues and are the exclusive β-receptor present on pulmonary vascular smooth muscle (Carstairs et al., 1985). β2-receptors in the epithelium contribute to alveolar fluid clearance, while those in smooth muscle promote bronchodilation (Mutlu and Factor, 2008). β1-receptors present onof the alveolar wall and submucosal glands (Carstairs et al., 1985) and contributes to alveolar fluid clearance (Sakuma et al., 2001), although not to the extent of β2 (Mutlu et al., 2004).

Adrenergic overstimulation has several pulmonary effects germane to critical illness concerns, including pulmonary edema and elevated pulmonary pressures with right heart dysfunction, most notably through α-receptor-mediated vasoconstriction (Dunser and Hasibeder, 2009). α-receptor-mediated vasoconstriction increases the displaced blood volume into the pulmonary circulation, increasing congestion and capillary wall stress. Pressure increase and fluid retention readily shift fluid into the pulmonary interstitium and the alveoli, especially when inflammation disrupts the capillary barrier. Although less influential than α stimulation, β1 stimulation can augment right ventricular output, further increasing pulmonary blood volume and pulmonary capillary pressures (Rassler, 2012). However, β2-agonism is often associated with improvements in mechanisms thatmay mitigate prevent edema throughsuch as alveolar fluid clearance (Maron et al., 2009). β2-agonism may produce other protective pulmonary effects such as reductions in systemic and pulmonary inflammatory cytokines (Maris et al., 2005; Bosmann et al., 2012) and prevent capillary permeability (de Prost et al., 2008). Clinical trials failed to translate pre-clinical evidence into positive outcomes as β2-agonism increased acute respiratory distress syndrome mortalitylinical trials in critical illness have failed to show positive outcomes as β2-agonism increased mortality from acute respiratory distress syndrome (Gao Smith et al., 2012). The lack of benefit may occur secondary to dysfunctional β2-receptors during prolonged inflammatory states (Giembycz and Newton, 2006; Belletti et al., 2020). Notably, β2-mediated vasodilation may detrimentally affect blood shunting in cardiopulmonary resuscitation leading to the distribution of blood from well to unventilated alveoli (Thrush et al., 1997). The mode of critical illness likely influences the degree of pulmonary pathophysiology with β-receptor stimulation, with insufficient evidence to malignCurrently, insufficient evidence exists to support a role for β2 stimulation as helpful or harmful in critical illness and possible harm in long-term pulmonary dysfunction like acute respiratory distress syndrome.

#### Pulmonary β-Blockade Effects

βB provides a potential strategy to improve pulmonary adrenergic response. Prescribing of βB typically warrants caution in pulmonary pathologies, most notably chronic obstructive pulmonary disease (COPD) and asthma, as βB can reverse the benefits of β2-mediated bronchodilation, although cardioselectivity β1 blocking agents eliminates some concern (MacNee, 2019). Nevertheless, Aa recent large clinical trial determined metoprolol use in COPD patients without cardiac indications for a βB resulted in increased exacerbations (Dransfield et al., 2019). However, in critically ill patients with acute respiratory failure and COPD, β1-selective βB did not affect ICU length of stay (Kargin et al., 2014). Additionally, continuing cardioselective βB for patients with underlying cardiac indications hospitalized for COPD exacerbations appears safe (Stefan et al., 2012). In asthma, several clinical and database studies have suggested that βB use does not worsen airway hyperresponsiveness or asthma exacerbations (Short et al., 2013; Morales et al., 2017). A network meta-analysis of 24 clinical trials concluded that non-selective βB (specifically oral timolol and propranolol infusions) were associated with a higher incidence of asthma attacks than β1-selective βB. MoreoverAdditionally, antecedent cardioselective βB therapy has been associated with lower mortality in ICU patients with acute respiratory failure, and βB withdrawal may worsen mortality (Noveanu et al., 2010). In a retrospective assessment of βB commencement after about 7 days of ICU admission, no alterations in pulmonary function occurred (Van Herpen et al., 2019). Given the current evidence, compelling cardiac indications (e.g., atrial fibrillation, ischemic heart disease) should drive βB use in critically ill patients, and COPD and asthma should not restrict βB use. given the current evidence.

Some preclinical evidence suggests possible protective mechanisms of βB germane to critical illness. Maccari et al. reported various selective and non-selective βB prevented catecholamine-induced β2 downregulation *in-vitro* (Maccari et al., 2020). Other pre-clinical studies demonstrate βB lung-protective effects in sepsis-induced acute lung injury. The ultra-rapid βB, landiolol, suppressed lung injury and reduced lung injury associated protein, high-mobility group box 1 (HMGB-1), in a rat lipopolysaccharide sepsis model (Hagiwara et al., 2009). The mechanism of pulmonary benefit of βB in these settings remains a conjecture, although when applied in clinical settings, the effects do not appear detrimental to pulmonary physiology.

### Disease-specific Evidence for β-Blockade

#### Sepsis

Dysregulated inflammatory response and catecholamine upregulation affect nearly every organ system in sepsis. Two specific derangements include hemodynamic compromise and metabolic alterations, which may open a role for βB (Plank et al., 1998; O'Dwyer et al., 2006; Furian et al., 2012). Sepsis leads to elevated serum pro-inflammatory cytokines (e.g.,TNF-α, IL-1β, and IL-6). Cytokine up-regulation has multiple deleterious effects, possibly mitigated by βB (Cain et al., 1999; Hsueh and Law, 2003). Indeed, βB have been proposed to reduce sepsis-induced cardiac dysfunction, improve the sepsis-induced hypermetabolic state, and play a role in immunomodulation by preventing lymphocyte apoptosis prevalent within inflammatory mechanisms of sepsis (Suzuki et al., 2017). Additionally, sepsis precipitates tachycardia, which reduces filling time and increases the risk of arrhythmias, potentially exacerbating the poor hemodynamics of impaired systemic vascular resistance (SVR) (Jacobi, 2002; Suzuki et al., 2017). Heart rate reduction via β1 blockade in the setting of adequate preload can decrease myocardial oxygen consumption, increase diastolic filling time, and increase coronary perfusion time, all potentially reducing the risk of myocardial ischemia and improvement in end-organ perfusion. β1 blockade may result in hemodynamically significant hypotension in patients without adequate preload and should therefore be used cautiously or avoided. The 2016 Surviving Sepsis Guidelines do not make recommendations regarding βB continuation or initiation in septic patients (Rhodes et al., 2017).

The hemodynamic improving effects of acute βB use in sepsis remain controversial; however, case series and small retrospective and prospective studies have established a plausible safety profile. As early as 1972, a case series in refractory septic shock patients documented the hemodynamic effects of propranolol (Berk et al., 1972). The cases conceptualized hyperdynamic vs. hypodynamic shock, given the observation that three patients dying after propranolol use had severely reduced CO compared to those who survived. Analysis of hemodynamic parameters continued in retrospective reviews of septic patients; however, unlike Berk et al., a study conducted by Schmittinger et al (Schmittinger et al., 2008) found no change in cardiac index (CI) following milrinone infusion with enteral metoprolol initiated after hemodynamic stabilization. HR control (65–95 bpm) was achieved in 39 out of 40 patients in addition to a significant increase in stroke volume index (SVI) (*p* = 0.002), and central venous pressure (CVP) along with dosages of norepinephrine, vasopressin, and milrinone all decreased (*p* < 0.001). Other small retrospective studies of βB in sepsis have not shown increases in mortality through acute βB use (Gutierrez et al., 2009).

Subsequent small prospective observational studies continued to analyze hemodynamic parameters following βB, specifically esmolol, in sepsis. Some studies demonstrated significantly decreased CO proportional to the decreases in HR (Gore and Wolfe, 2006) while others showed unchanged CO (Morelli et al., 2016) or insignificant reductions in CO (Balik et al., 2012). A more consistent trend was seen with regard to SV with evidence of significant (Du et al., 2016; Morelli et al., 2016) or negligible increases documented (Balik et al., 2012). In a subgroup analysis, Du et al. demonstrated that in patients with increased SV, esmolol therapy had a lower risk for decreased CO (Du et al., 2016). Similarly to Schmittinger et al. (Schmittinger et al., 2008), Morelli et al.(Morelli et al., 2016) observed reduced norepinephrine requirements after esmolol, although not all studies uniformly observed this difference (Balik et al., 2012). Measures of tissue perfusion, including lactate levels, were conflicting amongst studies, with some showing significant decreases in the esmolol group (Du et al., 2016) while others had more substantial reductions in the control group (Shang et al., 2016). The prospective studies did not analyze the risk or incidence of mortality associated with esmolol therapy, but Shang et al.(Shang et al., 2016) concluded a significantly shorter mechanical ventilation duration with esmolol compared to control (*p* < 0.05). Concerning the timing for the initiation of esmolol, these prospective studies were relatively consistent by attempting to correct preload through fluid resuscitation before administration of an esmolol loading dose. Nevertheless, timing, thresholds, and parameters for hemodynamic stabilization varied. These retrospective and prospective data are collectively limited by small sample sizes and lack relevant clinical outcomes, establishing impetus for larger randomized trials.

The seminal Morelli et al. (Morelli et al., 2013) phase 2 study of esmolol in septic shock patients requiring high-dose vasopressors revived discussion of βB in sepsis. Esmolol achieved the target HR (80–94 bpm) in all patients (−28 bpm; IQR = −37 to −21) compared to standard of care (−6 bpm; 95% CI = −14 to 0) and resulted in a mean reduction of 18 bpm (*p* < 0.001). The esmolol group exhibited improvements in SV and left ventricularLV stroke work index and decreases in norepinephrine and fluid requirements. Esmolol also improved pH, base excess, and arterial lactate. Several other randomized controlled trials (RCTs) evaluated βB in sepsis (Orbegozo Cortes et al., 2014; Yang et al., 2014; Wang et al., 2015; Xinqiang et al., 2015; Wang et al., 2017; Liu et al., 2019; Kakihana et al., 2020). Cumulatively, these trials have recently been assessed in systematic reviews and meta-analyses.

Chacko et al. evaluated 9 studies in a systematic review and found benefit from most studies with regards to heart rate control, decreased mortality, and acid-base parameters although strength of evidence is limited due to heterogeneity and inclusion of only one RCT (Chacko and Gopal, 2015). Sanfilippo et al. (Sanfilippo et al., 2015) was the next systematic review published that included two RCTs with the additional evidence from Yang et al. (Yang et al., 2014) At this time, the sizeable differences in sample size and trial design did not allow for a meta-analysis, but this systematic review further affirmed that βB use in septic and septic shock patients conferred decreased HR without significant adverse effects (Sanfilippo et al., 2015). The previous systematic reviews include a range of trial designs, but a meta-analysis conducted in 2018 evaluated the use of esmolol on septic shock and sepsis from five RCTs (Liu et al., 2018). The three trials that reported survival rate showed that esmolol use when compared to control was associated with a significantly increased rate of survival (RR = 2.06; 95% CI = 1.52–2.79; *p* = 0.006). With regard to hemodynamics, esmolol use showed no influence on MAP, CVP, or central venous oxygen saturation (ScVO_2_) but did reduce HR and cardiac biomarker troponin I. In 2019, Lee et al. (Lee et al., 2019) published a systematic review of 14 studies which included 5 RCTs, although only three of these RCTs were the same as those included in the Liu et al. meta-analysis. Six of the studies assessed βB use and mortality, which despite possible publication bias, demonstrated average odds ratio of 0.4072 (95% CI = 0.2602–0.6373) in favor of βB use.

Since the publication of these systematic reviews and meta-analyses there has been an increased focus on the treatment of tachyarrhythmias in sepsis. Initial evidence garnering support for βB use in septic patients with atrial fibrillation resulted from a 2016 propensity-matched cohort study. This analysis concluded that βB were associated with lower hospital mortality when compared to calcium channel blockers (CCBs), digoxin, and amiodarone (Walkey et al., 2016). With regard to rate control, Bosch et al. found that in comparison to CCBs, amiodarone, and digoxin, βB improved HR control to <110 bpm at 1 hour for the treatment of sepsis-associated atrial fibrillation, although this effect did not persist to show meaningful difference at 6 h (Bosch et al., 2020). While these studies included a variety of βB agents, newer evidence has shifted to solely focus on the use of ultra-short-acting βB, esmolol and landiolol. Of note, landiolol is not available for use in the United States. Kakihana et al. (Kakihana et al., 2020) analyzed the safety and efficacy of landiolol in a multicenter, open-label RCT in Japan that showed significant improvements in HR control and decreased incidence of new-onset arrhythmias. This trial specifically focused on a patient population with HR ≥ 100 bpm maintained for at least 10 min without a change in catecholamine dose and with a diagnosis of atrial fibrillation, atrial flutter, or sinus tachycardia. The most common adverse effect was hypotension, which quickly resolved in all instances given the ultra-short-acting nature of the drug. Hasegawa et al. performed a systematic review and meta-analysis of seven RCTs associated with esmolol and landiolol use in patients with persistent tachycardia (defined as HR > 95 bpm) despite initial resuscitation.(Hasegawa et al., 2021). Six of the RCTs included reported 28-days mortality. The use of ultra-short-acting βB in this patient population of 572 patients was found to be associated with significantly lower 28-days mortality (RR = 0.68; 95% CI = 0.54–0.85; *p* < 0.001) with an absolute risk reduction of 18.2% conferring a number needed to treat of 6 to prevent one patient death.

The use of beta-blockade in septic patients remains controversial especially with regard to timing of initiation. Tachycardia in the early stages of un-resuscitated sepsis is a major compensatory mechanism to ensure cardiac output, oxygen delivery, and perfusion. The use of beta-blockade, specifically with esmolol and landiolol, has been shown to reduce heart rate in the septic patients without deleterious effects on end-organ perfusion and may be associated with improved survival rates. Despite some dosing and timing variation within the RCTs that have been conducted, there is a general consensus that βB should not be initiated until at least 6 h, and in some trials 24 h, after the *initial* fluid resuscitation and vasopressor use. With this in mind, βB therapy may be initiated while patients are still requiring vasopressors. In fact, many studies described potential for decreased norepinephrine requirements with βB, instigating hypotheses of βB as vasopressor-sparing with potential to avoid deleterious effects of prolonged, high catecholamine requirements. Therefore, use of esmolol should be based on patient specific factors and likely should be considered only after initial resuscitation and once hemodynamic stabilization with vasopressors is achieved. Without large randomized controlled trials evaluating and elucidating the optimal dosing regimen and initiation timing considerations, the cost of esmolol infusion course should be considered as many hospital formularies restrict its use due to the extensive significant cost of the drug.

There are numerous retrospective studies that have investigated premorbid βB exposure prior to admission to the ICU with a diagnosis for sepsis that have conferred mortality benefit. Macchia et al. performed a retrospective observational study in 9,465 septic patients that concluded lower 28-days mortality in patients previously prescribed βB (Macchia et al., 2012). As part of a national cohort of Medicare beneficiaries, Singer et al. determined outpatient βB prescription was associated with a significantly reduced in-hospital and 30-days mortality, with no difference in regards to cardioselective compared to non-selective βB (Singer et al., 2017). In contrast, a recent observational cohort study by Guz et al. found that antecedent cardioselective βB were associated with a stronger protective effect on 30-days mortality rate reduction than noncardioselective βB for patients admitted with sepsis (Guz et al., 2021). Based on additional subgroup analyses according to tachycardia stratification, both patients with absolute and relative tachycardia on presentation exhibited reduced 30-days mortality rates with βB use.

Beyond initiation of βB in sepsis or premorbid βB use, continuation of chronic βB in patients admitted with sepsis and septic shock remains controversial, with common practice being to discontinue anti-hypertensive therapy upon admission. A prospective, observational study evaluated 296 patients admitted with severe sepsis or septic shock who were on chronic beta-blocker therapy (Fuchs et al., 2017). Chronic beta-blocker therapy was continued in 167 patients and was associated with significant decreases in hospital, 28-days, and 90-days mortality (*p* < 0.05) compared to βB cessation. Continuation of beta-blockade therapy was also associated with decreased crystalloid requirements during the first 24 h (*p* = 0.049) without increases in need for vasopressor, inotropic support, or low-dose steroids. To build on these results, a systematic review including a total of nine studies and over 6,500 patients found that premorbid beta-blocker exposure, regardless of continuation, in patients with sepsis was associated with reduced mortality (Tan et al., 2019). Although the precise mechanism of benefit in these settings is unknown, potential explanations beyond the mechanisms mentioned previously in this section include the prevention of rebound effects of tachycardia, hypertension, and arrhythmias caused by abrupt βB withdrawal.

In summary, Tthe hemodynamic evidence for βB use in sepsis has been proven as there are numerous studies demonstrating decreased HR without significant change in MAP, CVP, or ScVO_2_. Further, the recent evidence for ultra-short-acting βB, esmolol and landiolol, especially with regard to decreased incidence of arrhythmias and 28-days mortality benefit is clinically significant. In fact, some are realizing a need to stratify subgroups within septic cohorts based on the potential benefit of cardiovascular intervention to decrease the negative consequences of tachyarrhythmias (Morelli et al., 2020). The inconsistencies in terms of dosing and timing of initiation within the existing evidence require subsequent investigation in robust randomized controlled trials. Overall, esmolol was the most studied βB in sepsis, and initial doses varied over a wide range of either weight based dosing (most commonly 0.05 mg/kg/min) or standard dosing (most commonly 25 mg/h) with doses titrated to heart rate reductions of 20% or a heart rate goal of 70–100 bpm. Additionally, there is ample evidence to show antecedent βB use confers mortality benefit, but there is only one RCT evaluating the continuation of chronic βB therapy in acute sepsis, which warrants supplementation.

#### Burns

Severe burns lead to catecholamine release and a hypermetabolic state characterized by increased cardiac output, increased energy requirements, muscle breakdown, and general catabolism (e.g., reduced bone density, etc) (Wilmore et al., 1974; Herndon et al., 2001). This response lasts for at least 9 months and up to 2 years and is associated with a hypercatabolic state leading to muscle and bone loss (Hart et al., 2000; Przkora et al., 2006). β1 receptor mediated lipolysis and agonism of β2 receptors can cause glycogenolysis and gluconeogenesis within hepatocytes due to catecholamine stimulation (Novotny et al., 2009). Hypermetabolism can negatively impact the function of skeletal muscle, skin, and the immune system, ultimately resulting in multiorgan dysfunction and even death (Núñez-Villaveirán et al., 2015).

As such, βB are an attractive intervention to prevent the hyperadrenergic cascade that follows burn injury. Preclinical animal studies examining propranolol to improve wound healing following burn injury have noted enhanced wound healing and reduced activity of local inflammatory pathways (Romana-Souza et al., 2008; Zhang et al., 2009). Nearly all studies investigating βB in burn injuries have been conducted in pediatric patients using propranolol (Núñez-Villaveirán et al., 2015). Propranolol has been associated with a decrease in HR and oxygen consumption and the reversal of catabolism, evidenced by significant reductions in resting energy expenditure (REE) and prevention of lean body mass loss (Herndon et al., 2001; Jeschke et al., 2007; Williams et al., 2011; Herndon et al., 2012).

Baron et al. (Baron et al., 1997) deemed propranolol use safe and effective for ≥10 days following severe burns (≥40% of total body surface area [TBSA]) in 22 children aged 1–10 years old. In this population, propranolol use demonstrated significantly decreased HR and rate pressure product (RPP), defined as MAP multiplied by HR, without adverse effects. Herndon et al. (Herndon et al., 2001) extended this time frame to at least 2 weeks by evaluating propranolol in 25 pediatric burn patients (>40% of TBSA). Propranolol showed successful attenuation of the hypermetabolic response by decreasing REE, oxygen consumption, and muscle catabolism. Additionally, lean mass loss at 2 weeks was prevented by propranolol (9% loss vs 1% loss; *p* = 0.003). Similarly, Jeschke et al. (Jeschke et al., 2007) found improvements in REE with propranolol in 245 severely burned children. In a separate trial, Herndon et al. (Herndon et al., 2012) investigated propranolol given within 96 h from admission and continued for a year compared to control in 179 pediatric burn patients with burns >30% of TBSA. While there was no significant difference in mortality (*p* = 0.72), propranolol use did result in reduced cardiac work and improved lean body mass and bone density without adverse events. In patients receiving propranolol, the percent of predicted HR was significantly lower and persisted up to a year postburn; however, significant reductions in REE and RPP were only sustained through 6 months, while no difference was seen at 1 year.

A large clinical trial evaluated propranolol’s effects on cardiac function when started 24–72 h after admission versus control in 406 children with burns >30% of TBSA (Williams et al., 2011). CO decreased after 2 weeks of starting propranolol and the reduction continued throughout the trial. SV, when compared to non-burned children of the same age, was higher in patients receiving propranolol versus control (112 ± 8% vs. 94 ± 5%; *p* < 0.02), likely a function of the reduced HR allowing for longer ventricular filling times. RPP decreased in the group receiving propranolol, indicating lower myocardial oxygen consumption. These results suggest that propranolol has a significant hemodynamic impact on pediatric burn patients.

Data for βB use in burned adults are limited, but the available evidence supports conclusions comparable to these pediatric studies. Arbabi et al. (Arbabi et al., 2004) compared three cohorts: preexisting βB use continued during hospitalization, new hospital βB use, and no βB use in adult burn patients. Unlike the pediatric studies, βB selection varied with most receiving metoprolol, atenolol, and esmolol, and few receiving propranolol. Preexisting βB was associated with a significantly lower rate of mortality than βB initiation during hospitalization and no βB (5 vs. 27% and 13%, respectively). The higher mortality rate in the hospital-initiated βB group may be due to the presence of tachyarrhythmias treated with βB and more severe underlying disease, which was supported by prolonged ICU and hospital stays in the group. Preexisting βB was associated with a shorter mean healing time of 21 days when compared to control (*p* = 0.02). These data suggest antecedent βB use may confer a lower risk of mortality and accelerated healing time, which complements the data for improved outcomes in other adrenergic stress states like sepsis.

In 2009, Mohammadi et al. (Mohammadi et al., 2009) randomized 79 adult burn patients to propranolol or control and assessed wound healing dynamics. Patients receiving propranolol had more rapid healing times and reductions in required graft size (13.75 vs 18.75%; *p* = 0.006) in addition to shorter hospital length of stay (24.41 vs 30.95 days; *p* = 0.05). To build on these results, Ali et al. (Ali et al., 2015) evaluated the effect of propranolol on wound healing and blood loss in a cohort of 69 adult burn patients. Patients receiving propranolol initiated within 48 h of admission had a shorter recovery time with an average of 10 ± 5 days in between skin grafting procedures, whereas patients in the control group had an average of 17 ± 12 days in between procedures (*p* = 0.02). When hematocrit levels were drawn perioperatively, patients receiving propranolol showed a 5–7% increase compared to control (*p* = 0.002). Notably, the propranolol patients required larger grafts, but no differences in blood transfusions were observed, thus concluding the utility of propranolol for diminishing blood loss during skin graft procedures and improving wound healing. Further investigation in a recent Pakistani clinical trial of propranolol in 70 patients started day three post-burn demonstrated similar reductions in healing time (about a 1 week reduction) and time to graft readiness (10 days reduction) (Cheema et al., 2020). Propranolol also resulted in shorter hospitalization (26.7 vs. 33.6 days; *p* < 0.001).

Overall, the evidence suggest βB are effective in improving burn recovery in both pediatric and adult patients. By mitigating the adrenergic response at early time points after burns, βB can lessen the negative effects of the hyperadrenergic burn state. The 2012 American Burn Association (ABA) Burn Quality Consensus Conference Summary agreed that βB use is beneficial in pediatric and adult burn patients but recommended further research due to the lack of level one evidence at that time (Gibran et al., 2013). The International Society for Burn Injuries (ISBI) released the Practice Guidelines for Burn Care, Part 2 in 2018 with a recommendation to use a nonselective βB in burn patients ≤18 years old with the goal of reducing HR to 75% of the admission HR (Allorto et al., 2018). Since the publication of the ISBI guidelines in 2018, there has been no new evidence in pediatric burn patients; however, the Cheema et al. trial in Pakistan provides additional, robust evidence in adult burn patients which may lead to increased guidance in this population. While these guideline statements do make recommendations for βB use and monitoring including HR and weight loss, they do not specify timing or dosing. Based on the studies evaluated, propranolol initiated within one to 3 days after burn injury has the strongest evidence in both children and adults.

Overall, the evidence suggest βB are effective in improving burn recovery in both pediatric and adult patients. By mitigating the adrenergic response at early time points after burns, βB can lessen the negative effects of the hyperadrenergic burn state. Dosing evaluated in these studies with the strongest evidence in both children and adults was propranolol 1–3 mg/kg/day within one to 3 days after burn injury and titrated based on hemodynamic effects. Adults maintained on another βB agent may be better served continuing their current βB instead of switching to propranolol; however, no evidence has addressed head-to-head comparisons of βB providing an area for future research.

#### Traumatic Brain Injury

Following traumatic brain injury (TBI), a systemic hyperadrenergic state develops characterized by adrenal release of catecholamines and sympathetic activation (Clifton et al., 1981). The surge in catecholamine levels causes vasoconstriction, worsened cerebral ischemia, increased intracranial pressure, all leading to ongoing secondary injury to brain tissue (Lazaridis, 2017; Rizoli et al., 2017). βB can theoretically inhibit the catecholamine interaction with beta adrenergic receptors thus obstructing the detrimental sympathetic nervous system hyperactivity associated with severe TBI. Benefit may also be seen from βB by decreasing the cerebral oxygen demand, thus improving relative ischemia (Clifton et al., 1981).

Substantial pre-clinical evidence has collectively found that βB reduce cerebral ischemia and increase cerebral perfusion following traumatic insult (Goyagi et al., 2006; Ley et al., 2009; Goyagi et al., 2010; Iwata et al., 2010; Ley et al., 2010; Umehara et al., 2010; Goyagi et al., 2012; Song et al., 2014). Neurological deficit scores and infarct volumes were decreased in rats or mice that were treated with βB. Differences in the route of administration, agent chosen, dose, and timing varied but globally use of βB appears to confer benefit. Propranolol (Goyagi et al., 2006; Ley et al., 2009; Iwata et al., 2010; Ley et al., 2010), esmolol (Goyagi et al., 2006; Goyagi et al., 2010; Iwata et al., 2010; Umehara et al., 2010; Goyagi et al., 2012), landiolol (Goyagi et al., 2006; Goyagi et al., 2010; Iwata et al., 2010; Umehara et al., 2010; Goyagi et al., 2012), carvedilol (Goyagi et al., 2006), and betaxalol (Song et al., 2014) have all been investigated. Goyagi et al. (Goyagi et al., 2006) found no difference between intravascular versus intrathecal administration, Song et al. (Song et al., 2014) only investigated intraventricular administration, and all other studies used intravascular administration. Iwata et al. was the only study to indicate medication preference where esmolol and landiolol showed superior neuroprotection compared to propranolol in postischemic treatment. (Iwata et al., 2010). Higher doses of propranolol (4 mg/kg) were preferred to lower doses (1 mg/kg) (Ley et al., 2010), while no difference was observed amongst varying doses of esmolol and landiolol (Goyagi et al., 2012). Conflicting evidence exists for the timing of βB administration, where Ley et al. (Ley et al., 2010) observed that initiation of βB treatment pre-TBI was equivalent to post-TBI while Iwata (Iwata et al., 2010) found only post-TBI initiation benefit.

To date, only one RCT regarding beta-blocker use in TBI has been conducted by Cruickshank et al. (Cruickshank et al., 1987) Secondary to unclear randomization and allocation concealment method in addition to incomplete outcome data reported, the trial has largely been discounted due to a high risk of bias; however, it did show a positive correlation between arterial noradrenaline concentration and cardiac damage. (Cruickshank et al., 1987) (Alali et al., 2017) Additionally, fewer βB-group patients experienced supraventricular tachycardia (6 vs. 28; *p* < 0.0001) and ST-segment and T-wave changes (15 vs. 26; *p* = 0.062). βB use also inhibited further increases in myocardial isoenzyme of creatine kinase (CKMB) and abolished focal myocardial necrotic lesions compared to placebo. The remainder of the clinical evidence regarding βB use in TBI is from one multi-institutional, prospective, observational study and nine observational cohort studies, but overwhelmingly, this evidence concludes a mortality benefit for use of βB in TBI (Alali et al., 2017; Chen et al., 2017).

Within the nine retrospective cohort studies conducted, eight analyzed a primary outcome of in-hospital mortality (Arbabi et al., 2007; Cotton et al., 2007; Inaba et al., 2008; Schroeppel et al., 2010; Schroeppel et al., 2014; Mohseni et al., 2015; Ko et al., 2016; Murry et al., 2016; Zangbar et al., 2016). After adjustments, βB use after TBI was associated with statistically significant lower mortality in seven out of the eight studies with primary outcomes of in-hospital mortality (Arbabi et al., 2007; Cotton et al., 2007; Inaba et al., 2008; Schroeppel et al., 2010; Mohseni et al., 2015; Ko et al., 2016; Zangbar et al., 2016). Schroeppel et al. (Schroeppel et al., 2014) showed similar adjusted odds of mortality amongst all subjects, but subgroup analysis revealed lower odds of mortality in patients who received propranolol. The βB cohorts typically were comprised of older subjects (Arbabi et al., 2007; Inaba et al., 2008; Schroeppel et al., 2010; Mohseni et al., 2015) with more severe head injuries (Arbabi et al., 2007; Inaba et al., 2008; Schroeppel et al., 2010; Mohseni et al., 2015) as indicated by lower GCS levels (Arbabi et al., 2007; Ko et al., 2016) and therefore investigators adjusted for potential confounding factors. In-hospital mortality was a secondary outcome in Murry et al. (Murry et al., 2016) where no difference was observed, although no adjustments were made. A meta-analysis of all nine cohort studies, which included 8,245 patients, revealed a statistically significant mortality reduction when patients were exposed to beta-blockers after TBI (pooled OR = 0.39; *p* < 0.00001) (Alali et al., 2017). In all of the cohort studies, βB were initiated during hospital stay after the TBI had occurred and continued for varied durations. Two of the more recent studies from 2016 made an effort to administer propranolol earlier in the time course (within twelve (Murry et al., 2016) or twenty-four (Ko et al., 2016) hours of admission). Various βB were used amongst the studies with no preference cited between agents except in the aforementioned Schroeppel et al. study where propranolol reduced mortality compared to atenolol, carvedilol, esmolol, labetalol, metoprolol, and sotalol (Schroeppel et al., 2014). In 2017, based on a meta-analysis of these observational cohort studies, the Eastern Association of Surgery and Trauma (EAST), made a conditional recommendation for in-hospital use of βB in adults admitted to the ICU with severe, acute TBI and no contraindications to βB (Alali et al., 2017). The recommendation requires that hypotension (systolic blood pressure [SBP] < 90 mmHg) and symptomatic bradycardia (HR < 50 bpm) are avoided, but there is no formal recommendation on when to initiate βB, which βB to use, and how to titrate the βB therapy (Alali et al., 2017). In general, hypotension should warrant βB discontinuation or dose reduction.

In 2018, to build on the optimistic findings of these small single-center trials, the American Association for the Surgery of Trauma (AAST) conducted a multi-institutional, prospective observational trial (Ley et al., 2018). After analysis of 2,252 patients, the trial concluded that patients who received βB after TBI had a significantly lower adjusted (adjusted OR = 0.35; *p* < 0.001) and unadjusted mortality rate (13.8 vs. 17.7%; *p* = 0.013) in congruence with the 2017 EAST guideline recommendation. Further investigation revealed a reduction in mortality associated with propranolol use compared to all other βB (9.3 vs. 15.9%; *p* = 0.003). This study revealed no difference in neurological outcomes associated with βB use and patients who received propranolol had increased length of stay despite the aforementioned survival advantage.

NCT02957331, a randomized, open-label interventional trial, released study results on June 4, 2020 investigating the use of propranolol after TBI (Rizoli et al., 2017). The results show a difference of 7.7% propranolol arm versus 33.33% non-propranolol arm for all-cause 30-days mortality, although no analysis has been published. Propranolol was dosed to target a HR < 100 bpm and was held if the patient became hypotensive (SBP <100 mmHg) or bradycardic (HR < 60 bpm). The DASH After TBI trial (NCT01322048) is an ongoing, randomized, double-blind trial comparing propranolol and clonidine use to placebo (Patel et al., 2012). The primary outcome is ventilator-free days supplemented by multiple secondary outcomes, including all-cause mortality and neuropsychological outcomes. Interim data demonstrates decreased ventilator-free days and decreased percentage of all-cause mortality associated with adrenergic blockade (propranolol and clonidine) (Ley et al., 2009). No neuropsychological outcomes have been reported at this time. Only one propensity-matched case control study has addressed neuropsychological outcomes thus far, where beta-blockade was associated with shorter length of hospital stay and reduced risk of poor long-term functional outcome (Ahl et al., 2017).

In summary, βB use after TBI has been associated with decreased in-hospital mortality in one multi-institutional, prospective, observational trial, and nine retrospective cohort studies. Only one RCT has been conducted where mortality was not investigated; however, existing evidence supports the most recent 2017 EAST guideline recommendations of using βB following TBI. Studies evaluated a variety of both selective and nonselective βB in patients with a TBI; however, dosing was not reported in a majority of cases. Continued investigation in more robust trial designs may aid with clarification of preferred agent, dosing, titration, timing for initiation.

#### Cardiac Arrest

Epinephrine is part of the algorithm to treat pulseless ventricular tachycardia (VT) and ventricular fibrillation (VF) (Panchal et al., 2020); however, epinephrine, itself a catecholamine, can increase oxygen requirement of an already strained heart and may potentiate VF risk (Monroe and French, 1960). Thus, in addition to endogenous catecholamine release that can occur during ischemia, the cycle of catecholamine administration during VF may lead to refractory VF (RVF) or electrical storm (Nademanee et al., 2000; Lee et al., 2016). βB have been hypothesized to improve outcomes in VF by inhibiting elevated catecholamine levels to decrease myocardial oxygen demand and lower the threshold for VF (Lee et al., 2016). Animal studies have shown that βB have improved rates of resuscitation when used in cardiac arrest (Ditchey et al., 1994; Cammarata et al., 2004; Huang et al., 2004; Killingsworth et al., 2004). Several small trials evaluated the use of βB in refractory VF and electrical storm treatment and concluded that their use increases the rates of ROSC and overall survival (Nademanee et al., 2000; Skrifvars et al., 2003; Driver et al., 2014; Lee et al., 2016).

A small study evaluated the use of esmolol versus control on the incidence of sustained ROSC in patients with RVF (Lee et al., 2016). Sustained ROSC was greater in patients who received esmolol compared with those in the control group (56 vs. 16%; *p* = 0.007). Although there were significantly more patients who received esmolol that survived to ICU admission, survival and neurological outcome at 30 days, 3 months, and 6 months was not significant (*p* = 0.36). Similarly, Driver et al. (Driver et al., 2014) assessed the outcomes of 6 patients receiving esmolol versus 19 control patients who had RVF that started as VT or VF either outside of the hospital or in the emergency department. Patients in the esmolol group had greater incidence of sustained ROSC (67 vs. 32%) and survival to ICU admission (66 vs. 32%). Differing from the previous trial, patients receiving esmolol in this study had increased frequencies of survival to hospital discharge (50 vs. 16%) and discharge with fair neurologic outcome (50 vs. 11%), although these results were not statistically significant due to small sample size.

Nademanee et al. (Nademanee et al., 2000) studied the effects of sympathetic blockade in 27 patients vs. anti-arrhythmic agents in 22 patients with electrical storm. These patients had a myocardial infarction between 72 h and 3 months prior to developing electrical storm. Patients in the sympathetic blockade group received either propranolol, esmolol, or left stellate ganglionic blockade (LSGB). Because patients receiving beta blockers were analyzed in a combined group with those receiving LSGB, this limits our interpretation of the statistical analyses from the trial. No subgroup analysis of βB use alone was presented. Patients in the control group received lidocaine, procainamide, and/or bretylium tosylate as the anti-arrhythmic agent. Patients receiving a sympathetic blocker had a significantly higher survival rate at 1 week than patients who received an anti-arrhythmic (22 vs. 82%; *p* < 0.0001). Survival rate at 1 year was also greater in patients who received a sympathetic blocker versus an anti-arrhythmic (67 vs. 5%; *p* < 0.0001).

These three studies by Lee et al. (Lee et al., 2016), Driver et al. (Driver et al., 2014), and Nademanee et al. (Nademanee et al., 2000) were recently analyzed in a systematic review and meta-analysis by Gottlieb et al. (Gottlieb et al., 2020) Cumulatively, 115 patients were included with similar results to the individual studies of beta-blockade association with improved outcomes ranging from ROSC to survival with favorable neurologic outcome. The risk of bias was considered moderate to severe given the influence of confounding factors and selection of participants.

Propranolol was compared to metoprolol for electrical storm in combination with amiodarone for patients who had congestive heart failure and an implantable cardioverter-defibrillator (ICD) to assess the last VT or VF event that required the ICD for arrhythmia termination (Chatzidou et al., 2018). Patients receiving propranolol had 2.67 times fewer events of VT or VF (*p* = 0.001), as well as 2.34 times less incidences of ICD firings (*p* = 0.004). After 24 h, more patients receiving propranolol than metoprolol had not had an arrhythmia (*p* = 0.03). Propranolol was associated with higher likelihood of arrhythmia termination (*p* < 0.001), faster arrhythmia termination (*p* < 0.05), and decreased hospital length of stay when compared to metoprolol (*p* < 0.05). As such, non-selective β1 and β2 blockade appeared to result in more significant decreases in catecholamines and cardiac norepinephrine spillover leading to improvements in electrical storm control, whereas β1-selective blockers have been associated with increased cardiac norepinephrine spillover.

Metoprolol was also studied in patients resuscitated from out-of-hospital VF in a forward multiple logistic regression analysis to predict survival conducted by Skrifvars et al.(Skrifvars et al., 2003) Out of 102 patients total, 79 received beta-blocking agents (80%) which included the use of either metoprolol (intravenous or oral) or bisoprolol (oral). βB use during the first 72 h of post-resuscitation care was associated with survival at 6 months from the event in both the univariate (*p* < 0.001) and multiple logistic regression analyses (*p* = 0.002).

The 2017 AHA/ACC/HRS Guideline for the Management of Patients with Ventricular Arrythmias and Prevention of Sudden Cardiac Death (SCD) support the use of βB as first-line antiarrhythmic therapy for the treatment of ventricular arrhythmias and reducing the risk of SCD (Al-Khatib et al., 2018). Additionally, βB use is associated with a significant reduction in mortality in the setting of acute myocardial infarction (AMI) in addition to suppressing recurrent VF in patients with recent MI. The 2018 AHA Focused Update on ACLS Use of Antiarrhythmic Drugs During and Immediately After Cardiac Arrest does not recommend βB use immediately following cardiac arrest given limited evidence (Panchal et al., 2018). Upon review, esmolol 300–500 μg/kg loading dose followed by 0–100 μg/kg/min infusion was the most evaluated βB in the cardiac arrest studies, but propranolol, bisoprolol and metoprolol at variable doses were additionally studied. There is also some controversy as one study used a loading dose of esmolol 300–500 μg/kg while another studied esmolol 300–500 mg/kg accounting for a substantial one-thousandfold difference.

In summary, Tthe demonstration of improved rates of ROSC and sustained outcomes in addition to increased survival from βB (most notably with esmolol) use in patients with RVF is promising; however, larger studies are necessary to offer increased guidance on βB use during and after cardiac arrest in the coming years. Furthermore, additional research is needed to compare specific βB agents in cardiac arrest to build on existing evidence that non-selective agents may lead to fewer arrhythmias, improved arrhythmia termination, and decreased hospital length of stay when compared to β1-selective βB. However, once hemodynamic stabilization is achieved, current evidence is in line with guideline recommendations to initiate βB therapy to reduce risk of repeated VF (Al-Khatib et al., 2018).

## Conclusion

Although negative inotropes appear counterintuitive in hemodynamically compromised critical illness, dampening catecholamine signaling may confer a wide range of benefits, dependent on etiology. In Sepsis, immediately post fluid resuscitation and initial stabilization, re-initiation of home βB therapy should be strongly considered. Additionally, existing evidence suggests βB use may improve recovery following burn injury, reduce mortality rate in TBI, and increase achievement of ROSC in RVF cardiac arrest. Further, promising new data in sepsis suggest a potential role as well as further inquiry.
